# Patterns of Chemsex Substance Use and Its Association with HIV Transmission Risk Among Men Who Have Sex with Men in Thailand: A Latent Class Analysis

**DOI:** 10.1007/s10508-024-02868-8

**Published:** 2024-08-16

**Authors:** Doug H. Cheung, Nattharat Samoh, Kai J. Jonas, Sin How Lim, Yamol Kongjareon, Thomas E. Guadamuz

**Affiliations:** 1 Center of Excellence in Research on Gender, Sexuality and Health, Faculty of Social Sciences and Humanities, Mahidol University, Nakhon Pathom, 73170 Thailand; 2 Faculty of Tropical Medicine, Mahidol University, Bangkok, Thailand; 3 Department of Work and Social Psychology, Maastricht University, Maastricht, The Netherlands; 4 Department of Social and Preventive Medicine, University of Malaya, Kuala Lumpur, Malaysia; 5 John F. Kennedy School of Government, Harvard University, Cambridge, MA USA

**Keywords:** HIV risks, Men who have sex with men, Thailand, Substance use, HIV prevention, Sexual orientation

## Abstract

Sexualized substance use or “chemsex” may contribute to the HIV epidemic among men who have sex with men (MSM) in Thailand. Specific patterns of chemsex use may be associated with different HIV/STI transmission risks. We examined typologies and correlates of sexualized substance use among a sample of high-risk MSM (*n* = 532) who had attended a private sex party or circuit party in the past three years using latent class analysis. Multinomial regression was used to adjust for variables associated with the 3-class latent class model. We identified a 3-class model as the best fitting model, which included: (1) “negligible sexualized substance users” (36.7%), who had never engaged in chemsex in the past six months; (2) “sexualized substance users” (13.9%), who had a high level of sexualized alcohol use and a wide range of other substances before or during sex in the past six months; and (3) “exclusive chemsex users” (49.4%), who predominantly used crystal methamphetamine, amyl nitrite, and erectile dysfunctional drugs before or during sex in the past six months. Compared to negligible sexualized substance users, exclusive chemsex users were more likely to be HIV-positive, more likely to have six or more sexual partners in the past six months, less likely to have condom use at last anal intercourse, and more likely to have provided sex work. Relative to negligible sexualized substance users, sexualized substance users reported more frequent online sex-seeking behaviors. These subgroups of Thai MSM have unique substance use patterns and HIV-related risk profiles, underscoring the importance of targeted HIV prevention strategies.

**Supplementary Information:**

The online version contains supplementary material available at 10.1007/s10508-024-02868-8.

## Introduction

In Thailand, men who have sex with men (MSM) have a disproportionately high incidence of HIV, compared to the general population (Seekaew et al., 2018; Van Griensven et al., 2013, 2022). Epidemic trends in HIV infection indicate high rates of new infections among young MSM, those who use methamphetamines and other stimulant drugs, and those who sell or exchange sex (Dunne et al., 2019; Holtz et al., 2019; Piyaraj et al., 2018; Van Griensven et al., 2022). Sexualized substance use or “chemsex” among MSM may contribute to the HIV epidemic among MSM in Thailand (Kelly-Hanku, 2021). MSM engaging in sexualized substance use often engage in condomless anal sex, which increases their vulnerability to HIV transmission (Kelly-Hanku, 2021; Maviglia et al., 2022; Wang et al., 2023; Wong et al., 2020). This heightened risk of exposure to HIV is further exacerbated by the potential for multiple sexual partners and group sex during chemsex sessions (Lim et al., 2015; Scholz-Hehn et al., 2022; Wong et al., 2020) as well as an increased likelihood of having a sexually transmitted infection (STI) (Achterbergh et al., 2020; Lim et al., 2015; Scholz-Hehn et al., 2022; Tan et al., 2021; Wong et al., 2020). Additionally, the use of drugs such as methamphetamine can lead to prolonged sexual activity, thereby increasing the duration and intensity of potential HIV exposure (Semple et al., 2009). The combination of these factors creates a perfect storm for the rapid spread of HIV among MSM engaged in chemsex in Asia, highlighting the urgent need for targeted interventions to address this critical public health concern (Van Griensven et al., 2017).

Specific combinations of substance use may lead MSM to heighten their risk of HIV and STI transmission (Achterbergh et al., 2020; Melendez-Torres et al., 2018; Scholz-Hehn et al., 2022; Tan et al., 2021; Wong et al., 2020). For example, previous studies have found that MSM having a more intense and broader range of usage of substances, particularly the use of stimulant drugs, were more likely to report living with HIV and engage in condomless anal sex (Achterbergh et al., 2020; Melendez-Torres et al., 2018; Scholz-Hehn et al., 2022; Tan et al., 2021; Wong et al., 2020). Delineating the specific patterns of drug use can shed light on MSM’s differing risk levels, to which tailored interventions can be developed to reduce harm. For example, Wong et al. found that MSM belonging to latent classes of “medium threshold” and “intense users” were more likely to take preexposure prophylaxis (PrEP) or intend to use PrEP than other drug user types. Using latent class analysis (LCA) allows researchers to identify distinct subgroups or classes within a sample based on shared characteristics or behaviors (Lanza & Rhoades, 2013). The social hierarchy and power dynamics within the subculture of sexualized substance use and chemsex among Thai MSM could expose them to different sexual risks. For example, ice parties (crystal methamphetamine is referred to as “ice” in Thai) or “hi fun” (chemsex group session) is associated with a higher social class status and elite social network among Thai MSM as opposed to “yaba” (amphetamine tablets used by Southeast Asian blue-collar workers) (Guadamuz & Boonmongkon, 2018). An LCA approach can be used to determine the heterogeneity among these socially patterned sexualized substance use patterns to identify subgroups of Thai MSM who may have unique patterns of sexualized substance use. It is especially crucial to include rare but unique substances utilized by Thai MSM to inform the construction of a more culturally nuanced LCA model, as previous studies on Asian MSM utilizing the same latent indicator variables tended to reproduce results from Europe and North America (Guerras et al., 2021; Lim et al., 2015; Tan et al., 2021; Wong et al., 2020).

This study sought to understand the typology of chemsex patterns among a sample of Thai MSM who had attended a private sex party or a circuit party in the past three years. Private sex parties and circuity parties are social venues where the use of substances is prevalent (Colfax et al., 2001; Knox et al., 2020; Meunier, 2018). Circuit parties are large-scale events primarily geared toward the gay community, characterized by multi-day dance parties held in metropolitan areas with a high density of MSM. These parties are characterized by sexualized music, sexualized performances, naked bodies, and opportunities for sexual encounters (Cheung et al., 2015). In recent years, circuit parties have gained significant popularity in Asia, with attendance growing from a few thousand participants per night in 2007 to claims of over 25,000 attendees per night in 2023 (Cheung et al., 2015; Gcircuit Songkran 2024, n.d.; White Party Bangkok 2024, n.d.). On the other hand, our team’s previous qualitative research described the socioecological risks of “ice” parties where sexual orientation, unprotected sex, and ice use were enmeshed by a social pattern of desire, self-worth, and group conformity (Guadamuz & Boonmongkon, 2018). These social venues have been identified as high HIV risk environments due to prevailing social norms that discourage condom use negotiation and disclosure of HIV serostatus (Cheung et al., 2015; Colfax et al., 2001; Meunier, 2018; O’Byrne & Holmes, 2011). The current study sought to understand the sociodemographic and behavioral characteristics and patterns of chemsex usage of Thai MSM engaged in these social venues to inform tailored interventions.

## Method

### Participants and Procedure

A cross-sectional anonymous online self-administered survey (*n* = 532) was conducted. Inclusion criteria included men who were at least 18 years old, attended private sex parties (group sex of more than three persons), attended one of the circuit parties offered in Thailand (e.g., White Party, Gcircuit party, XXO Party) in the past three years, had anal intercourse with a man in the past year, ability to read Thai, and currently residing in Thailand. Data were collected from September to December 2021, and participants were recruited with the assistance of community-based partners and social media platforms (private Line® chat groups, private Facebook® groups, and private Twitter® groups). A web-based survey was delivered using Qualtrics software (Qualtrics headquarters, Australia [www.qualtrics.com]). To ensure the quality of data and prevent fraudulent entries, only relevant online groups were sent invitation links to the survey, and data validation features from Qualtrics (2024) were employed in flagging possible fraudulent responses, such as abnormal response speed, defined as more than two standard deviations from the median duration, and non-human activities. Upon completion of the survey, participants received incentives through a care package that included syringes, alcohol pads, condoms, and water-based lubricants. Informed consent was obtained from all participants after they reviewed the participant information sheet detailing risks and benefits of study participation, and then clicked “consent” to the online survey.

### Measures

Data on age, employment status, education, income, and sexual orientation were collected along with self-reported HIV-related risk behaviors. Participants were asked how many casual sex partners they had anal sex within the past six months; participants were allowed to choose a drop-down list ranged 0–100; and responses were categorized based on distributional features. Participants were also asked whether they had used a condom at last anal intercourse and the frequency of their participation in group sex (“often,” “occasionally” or “never”). Participants self-reported their HIV testing behaviors, HIV status, and STI diagnoses (gonorrhea, genital warts, genital herpes, syphilis, hepatitis B (HBV), hepatitis C (HCV), chlamydia, and pubic lice symptoms). Those reporting any of the above diagnoses were categorized as having an STI (excluding HIV). Participants were asked whether they had been given or received things or opportunities (e.g., mobile phones, money, clothes, bags, grades, or educational/work opportunities) in exchange for sex. They were also asked whether they had ever agreed to meet people from the Internet (Facebook) or geo-location social networking applications (apps) (Hornet, Jack’D) to have sex. Among those reporting a negative HIV status, participants were asked whether they had heard of PrEP, had taken PrEP, and whether they wanted to take PrEP.

Participants were asked whether they had ever used substances before or during sex in the past six months based on a list of options reported in our previous research (Guadamuz & Boonmongkon, 2018; Kongjareon et al., 2022). These options include amphetamine (“Yaba”/“Yama”), crystal meth (“Ice”), ecstasy, ketamine, GHB (gamma-hydroxybutyrate), Xanax/alprazolam, mephedrone, cocaine, heroin, opium, marijuana, amyl nitrite (“poppers”), erectile dysfunction medication, Kratom cocktail (“Fatal 4 × 100”), hormone medication (steroids, progesterone, estrogen), alcohol, and “never use substance before/during sex.”

### Data Analysis

LCA was used to identify patterns of substance use before or during sex (chemsex) among Thai MSM who attended a private sex party or circuit party in the past three months. A bias-adjusted classify–analyze approach was implemented in the poLCA package in R (Bray et al., 2015; Linzer & Lewis, 2011; Nylund-Gibson et al., 2019). First, an LCA model with only the latent class indicators (chemsex substance use variables) was fitted to determine the optimal number of latent classes based on the model fit criteria and theoretical interpretability. Models with two to six classes were evaluated with 20 random starts and a maximum of 5000 iterations per model. Model selection was based on log-likelihood (LL), approximate weight of evidence criteria (AWE), Bayesian information criteria (BIC), sample size-adjusted BIC (aBIC), and consistent Akaike information criteria (CAIC), with better fit indicated by higher LL and AWE values and lower BIC, aBIC, and CAIC values. A relative entropy above 0.70 provided evidence of adequate class separation as a model diagnostic. After identifying the optimal class solution, covariates were included in a second LCA model to derive bias-adjusted posterior probabilities that accounted for the relationships between covariates and latent classes. The participants were assigned to latent classes based on bias-adjusted posterior probabilities. Finally, multivariable multinomial logistic regression was used to relate participant characteristics to latent class membership, purposefully selecting covariates based on univariate analyses and theoretical relevance.

## Results

Sociodemographic and behavioral characteristics are summarized in (Table 1). The distribution of age, educational attainment, and prevalence of HIV testing behaviors resemble other studies involving Thai MSM recruited from a gay sauna and over the Internet (Khawcharoenporn et al., 2019; Weiss et al., 2017). However, our participants tended to earn a lower income and have a higher self-reported prevalence of STI diagnosis (59.2%) and HIV (20.3%) than other samples of Thai MSM (Khawcharoenporn et al., 2019; Seekaew et al., 2018). Our participants also had higher rates of condomless anal sex and reported higher numbers of casual sex partners than other Thai MSM (Khawcharoenporn et al., 2019; Weiss et al., 2017). Moreover, less than half of participants (44.7%) had received things or opportunities in exchange for sex. In comparison, more than half (54.7%) had provided things or opportunities in exchange for sex at elevated rates compared to representative Thai MSM samples (Seekaew et al., 2018). Almost all participants reported meeting their sexual partners online (97.2%). These results correspond to our qualitative findings on personal accounts of sexual transactions among ice party attendees (Guadamuz & Boonmongkon, 2018). Participants also reported a high willingness for PrEP use; among those without a positive HIV status, most of them had heard of PrEP (88.9%), 25.9% had taken PrEP, and among those who had not taken PrEP and had heard of PrEP, 83.1% wanted to take PrEP.

**Table 1:** Sociodemographic and behavioral characteristics of Thai MSM who attended a private sex party or circuit party in the past 3 years (*N* = 532)

	N	(%)
*Age* (Mean = 32.8, SD = 8.7)		
18–29	208	39.1
30–39	213	40.0
40 +	111	20.9
*Employment*		
Full time	303	57.0
Part time	74	13.9
Student/Unemployed/Others	155	29.1
*Education*		
None—Secondary	146	27.4
University/Tertiary	312	58.6
Postgraduate/Professional	74	13.9
*Monthly Income*		
Less than 100 USD	54	10.2
101–450 USD	222	41.7
More than 450 USD	256	48.1
*Sexual orientation*		
Gay	485	91.2
Bisexual	47	8.8
*Attended a private sex party or circuit party in past 3 years*		
Private sex party	474	89.1
Circuit party	58	10.9
*Ever tested for HIV*		
Ever	438	82.3
Never	94	17.7
*HIV Status*		
Negative	301	56.6
Positive	108	20.3
Don’t know	123	23.1
*Any STI in the past 12 months (excluding HIV)*		
Yes	314	59.2
No	216	40.8
*Number of sexual partners in the past 6 months*		
0–1	104	19.5
2–5	291	54.7
6 +	137	25.8
*Condom use in last anal intercourse*		
Yes	185	34.8
No	347	65.2
*Ever had group sex*		
Never	15	2.8
Sometimes	389	73.1
Often	128	24.1
*Ever received things or opportunities in exchange for sex*		
Yes	238	44.7
No	294	55.2
*Ever provided things or opportunities in exchange for sex*		
Yes	291	54.7
No	241	45.3
*Ever meet sexual partners online*		
Never	15	2.8
Occasionally	242	45.5
Often	275	51.7
*Heard of PrEP*		
Yes	377	88.9
No	47	11.1
*Taken PrEP*		
Yes	110	25.9
No	314	74.1
*Want to take PrEP*		
Yes	261	83.1
No	53	16.9

Most participants reported using at least one of the substances listed in (Table 2) with only 9% of the participants reporting never using any substance before or during sex. The prevalence of methamphetamine (40.2% by injection and 23.9% by inhalation) was the highest reported among studies on Thai MSM (Dunne et al., 2019; Fongkaew et al., 2022; Piyaraj et al., 2018; Thienkrua et al., 2018; Wansom et al., 2020). The most common substance used before or during sex in the last six months was amyl nitrite (45.9%), followed by the injection of crystal meth (40.2%). Alcohol use was moderate (29.9%), while erectile dysfunction medication (25.6%) was less prevalent than in another study among young Thai MSM in Bangkok (Thienkrua et al., 2018). Kratom cocktail use was 11.3%. Ecstasy, ketamine, GHB, Xanax, marijuana, and hormone medication usage were less frequently reported by participants. These characteristics of our participant sample reflect our recruitment strategy and study objectives in characterizing circuit party and private sex party attendees (Table 3).

**Table 2:** Substance use before or during sex in the last 6 months among Thai MSM who attended a sex party or circuity party in the past 3 years

	*N*	*(%)*
Amphetamine (“Yaba”/“Yama”)	21	3.9
Crystal Meth/“Ice” (Inhaling/Smoking)	127	23.9
Crystal Meth/“Ice” (Injecting)	214	40.2
Ecstasy	7	1.3
Ketamine	12	2.3
GHB	13	2.4
Xanax/Alprazolam	24	4.5
Mephedrone	0	0
Cocaine	2	0.4
Heroin	2	0.4
Opium	0	0
Marijuana	16	3.0
Amyl nitrite / “poppers”	244	45.9
Erectile dysfunction medication	136	25.6
Kratom cocktail/ “Fatal 4 × 100”	60	11.3
Hormone medication (steroids, progesterone, estrogen)	11	2.1
Alcohol	159	29.9
Never use substance before/during sex	48	9.0

**Table 3:** Prevalence and posterior probabilities for the inclusive 3-class LCA model of substance use during sex patterns among Thai MSM who attended a private sex party or circuit party in the past 3 years

	Negligible sexualized substance users	Sexualized substance users	Exclusive chemsex users
	36.1%, *ρ*	13.7%, *ρ*	50.2%, *ρ*
Amphetamine (“Yaba”/”Yama”)	0.00	0.22	0.02
Crystal Meth/”Ice” (Inhaling/Smoking)	0.00	0.09	0.45
Crystal Meth/”Ice” (Injecting)	0.07	0.22	0.69
Ecstasy	0.00	0.01	0.02
Ketamine	0.00	0.01	0.04
GHB	0.00	0.00	0.05
Xanax/Alprazolam	0.00	0.16	0.05
Cocaine	0.00	0.01	0.00
Heroin	0.00	0.03	0.00
Marijuana	0.00	0.11	0.03
Amyl nitrite / “poppers”	0.31	0.17	0.65
Erectile dysfunction medication	0.09	0.19	0.40
Kratom cocktail/ “Fatal 4 × 100”	0.00	0.79	0.00
Hormone medication (steroids, progesterone, estrogen)	0.00	0.10	0.01
Alcohol	0.22	0.76	0.23
Never use substance before/during sex	0.25	0.00	0.00

*ρ* = posterior probability estimated from latent class model

Based on the fit indices (see Supplementary Table 1) and respective elbow plots (see Supplementary Fig. 1), a 3-class model was supported by its lowest BIC and CAIC, with the lowest AWE among models with a number of parameters at 50 or higher, suggesting it to be the best fitting model. The 3-class model was refitted with covariates (the bias-adjusted inclusive 3-class model), which has improved LL, BIC, and RE, indicating that it provides a better fit and less classification uncertainty than the non-inclusive 3-class model. In the inclusive 3-class model (see Table 4), 36.7% (*n* = 195) were classified as “negligible sexualized substance users,” 13.9% (*n* = 74) were classified as “sexualized substance users,” and 49.4% (*n* = 263) were classified as “exclusive chemsex users.” Members of the “negligible sexualized substance users” subgroup reported the highest probabilities of never using any substance before or during sex, with 31% endorsing poppers and 22% endorsing alcohol before or during sex. Members in the “sexualized substance users” subgroup reported 9% and 22% of inhaling and injecting crystal meth and a variety of other substances (ranged 1%-19%) before or during sex, including Xanax, marijuana, amyl nitrite, erectile dysfunction medication, and hormone medication, and have the highest probabilities of using Kratom cocktail (79%) and alcohol (76%) before or during sex. “Exclusive chemsex users” subgroup had the highest probabilities (45% and 69%) of reporting both inhalation and injection of crystal meth before or during sex, the highest probability of using poppers (65%) and erectile dysfunctional drugs (40%) before or during sex, and low probabilities of reporting other substances before or during sex.

**Table 4:** Multivariable multinominal logistic regression examining participants’ sociodemographic and behavioral characteristics in relation to substance use before or during sex class membership (reference = negligible sexualized substance users)

	Sexualized substance users		Exclusive chemsex users	
	AOR	(95% CI)	AOR	(95% CI)
*Age*				
18–29	Ref		Ref	
30–39	0.91	(0.34–2.44)	4.53	(2.50–8.21)
40 +	0.29	(0.08–1.03)	1.35	(0.70–2.57)
*Education*				
None—Secondary	Ref		Ref	
Tertiary or above	0.14	(0.05–0.36)	1.87	(0.98–3.58)
*Attended a private sex party or circuit party in past 3 years*				
Private sex party	Ref		Ref	
Circuit party	0.32	(0.04–2.63)	4.58	(1.74–12.1)
HIV status				
Negative	Ref		Ref	
Positive	0.32	(0.04–2.63)	3.00	(1.56–5.79)
Don’t know	1.11	(0.42–2.96)	0.46	(0.24–0.89)
*Number of sexual partners in the past 6 months*				
0–1	Ref		Ref	
2–5	1.41	(0.32–6.24)	0.94	(0.51–1.74)
6 +	2.34	(0.45–12.3)	5.79	(2.48–13.5)
*Condom use in last anal intercourse*				
Yes	Ref		Ref	
No	3.39	(1.05–11.0)	3.91	(2.33–6.57)
*Ever received things or opportunities in exchange for sex*				
Yes	9.43	(3.01–29.6)	1.36	(0.75–2.47)
No	Ref		Ref	
*Ever provided things or opportunities in exchange for sex*				
Yes	6.87	(1.85–25.4)	5.01	(2.80–8.97)
No	Ref		Ref	
*Ever meet sexual partners online*				
Never to occasionally	Ref		Ref	
Often	17.2	(4.77–61.7)	1.08	(0.63–1.83)

McFadden’s pseudo-R^2^ = 0.453

AOR = adjusted odds ratio, CI = confidence interval

In our final multivariable model (see Table 4), compared to “negligible sexualized substance users,” “exclusive chemsex users” were more likely to be aged 30–39, compared to those aged 18–29, and self-reported HIV-positive status, compared to those self-reported to be HIV-negative, were more likely to have six or more sexual partners in the past six months, compared to having 0–1 sexual partner, and more likely to have ever provided sex work and more likely to not use a condom during last anal sex. “Exclusive chemsex users” were less likely to not know their HIV status compared to those self-reported to be HIV-negative. Compared to “negligible sexualized substance users,” “sexualized substance users” were more likely not to use condoms during last anal intercourse, more likely to have ever received or provided things or opportunities in exchange for sex, and more likely to report “often” to ever meet sexual partners online. For univariate analyses, please refer to Supplementary Table 2.

## Discussion

From a sexually active sample of Thai MSM who attended private sex parties or circuit parties in the past three years, we identified three distinct substance use typologies: “negligible sexualized substance users,” “sexualized substance users,” and “exclusive chemsex users” with differing levels of HIV and STI transmission risks. Those in the “exclusive chemsex users” group were more likely to be HIV-positive, have ever provided things or opportunities in exchange for sex, have a higher number of sexual partners, and report more inconsistent use of condoms. Corroborating evidence shows MSM that were classified into latent classes by endorsing any methamphetamine use were associated with HIV positivity, a higher number of sexual partners, condomless anal sex, and group sex (Achterbergh et al., 2020; Blair et al., 2022; Card et al., 2023; Goldshear et al., 2023; Lim et al., 2015; Melendez-Torres et al., 2018; Tan et al., 2021; Wong et al., 2020). The sexualized substance users characterized in the “exclusive chemsex user” class is indicative of the “ice party” subculture, where methamphetamine is predominately used to enhance and prolong sexual arousal and performance and to have sex with multiple partners (Guadamuz & Boonmongkon, 2018; Wang et al., 2023). Erectile dysfunction drugs are used to maintain erection, whereas amyl nitrite is used to facilitate penetrative anal sex (Guadamuz & Boonmongkon, 2018). This combination of sexualized substance use is associated with a heightened risk of HIV positivity, and among those not diagnosed with HIV, they could be exposed to substantial HIV risks (Fisher et al., 2010; Swartz & McCarty-Caplan, 2018). Men in this latent class membership may be aware of their heightened HIV risk and are more likely to be engaged in protective behaviors; thus, HIV-negative and sero-unknown participants who were classified in this latent class membership were more likely to be HIV tested, heard of PrEP, and have taken PrEP. This finding is consistent with studies examining chemsex prevalence and PrEP awareness among MSM in Malaysia (Maviglia et al., 2022) and Hong Kong (Wong et al., 2020). However, only 25.9% of our MSM sampled had ever taken PrEP, which is higher than previously reported by a study among Thai MSM engaged in sex work (Weir et al., 2022), but still far from UNAIDS’s 2030 goal of providing 50% PrEP coverage for high-risk MSM (UNIADS, 2022).

Those classified as “sexualized substance users” reported high levels of alcohol use and moderate levels of usage of a range of other substances. These substances included those typically seen in Europe and the USA, such as marijuana, amphetamine, and benzodiazepine, as well as substances unique to the Southeast Asian context, such as the Kratom cocktail and hormone medications. Kratom cocktails contain boiled Kratom leaves, cola soft drinks or iced tea, codeine, and a range of substances that could include mosquito coils, anxiolytic drugs, and antidepressants (Tungtananuwat & Lawanprasert, 2018). Together, these ingredients produce sedative and euphoric effects similar to alcohol consumption. Hormone medications, such as anabolic androgenic steroids, are used to boost physical performance and libido (Zaami et al., 2021). They are used to overcome unwanted side effects of methamphetamine, such as erectile dysfunction (Zaami et al., 2021). MSM in this subgroup could be using androgenic steroids to replace erectile dysfunctional medication due to over-the-counter accessibility of androgenic steroids. However, the use of anabolic androgenic steroids has been associated with violent behavior, which may contribute to the worsening epidemic of intimate partner violence among MSM (Parent & Johnson, 2023; Zaami et al., 2021).

Moreover, men classified into the “sexualized substance users” group tended to be part-time employed, less educated, and earning less income. These data indicate that these men are of a different socioeconomic class than the “negligible sexualized substance users” and “exclusive chemsex users” groups. This is consistent with our qualitative finding that those who uses “yaba” (amphetamine tablets) have a lower socioeconomic status (Guadamuz & Boonmongkon, 2018). Unlike “exclusive chemsex users,” these men are more likely to have never been tested for HIV, have an unknown HIV status, and are less likely to have heard of PrEP, possibly compounded by a lower socioeconomic status and decreased exposure to community-based resources such as HIV testing and PrEP awareness campaigns. In addition, these men could be relatively less aware of their HIV risk and face higher levels of barriers to accessing preventive and healthcare resources than “exclusive chemsex users” [16,17]. HIV risk perception is essential for engagement in HIV testing and acceptance of PrEP (Guadamuz et al., 2015; Khawcharoenporn et al., 2019; Plotzker et al., 2017). Public health interventions, such as voluntary HIV testing and PrEP promotion targeting this group of MSM, should raise awareness of their HIV risk perception to facilitate their acceptance of utilizing these services. Our data show that these men frequently meet sexual partners online; interventions can reach this group of men via online campaigns on geo-location social networking apps such as GRINDR, Jack’D, and Hornet (Guadamuz et al., 2015; Weiss et al., 2017).

Among our sample of Thai MSM, we found a very high prevalence of crystal meth use before or during sex (23.9% inhaled and 40.2% injected). The use of this chemical substance is among the highest in Asia (Wang et al., 2023). This finding corresponds to our recruitment strategy, which uses private sex parties and circuit parties as inclusion criteria, where substance use is the norm (Guadamuz & Boonmongkon, 2018; O’Byrne & Holmes, 2011). Nearly half of our sample of men had ever received things or opportunities in exchange for sex (44.7%) and more than half had provided things or opportunities in exchange for sex (54.7%). These factors are characteristic of chemsex epidemics in Asia (Maviglia et al., 2022; Wang et al., 2023). The power differential that occurs during transactional sex may further facilitate condomless anal sex (Dunne et al., 2019; Tan & Melendez-Torres, 2016; Weir et al., 2022). The combined social norm and transactional nature of these men’s engagement with chemsex may further subjugate their self-efficacy in negotiating condom use (Liu et al., 2009). Remarkably, only a quarter of the HIV-negative men sampled had taken PrEP, while 83.1% of those who had not taken PrEP wanted to take PrEP. This demonstrates the unmet need for PrEP coverage in high-risk men. Tailored interventions to promote PrEP uptake are urgently required.

### Limitations

Despite the importance of these findings, this study had some limitations. Our sample was drawn from a convenience sample of community partners. Although our study’s sociodemographic characteristics resemble those of other studies with similar recruitment strategies, they may not represent the true population of Thai MSM who attend private sex parties and circuit parties. They may have more distinct characteristics in terms of sociodemographic characteristics, risk behaviors, and substance use patterns that may extend the relationship between latent class and other covariates. We attempted to account for this heterogeneity by including the circuit party/sex party attendance variable in the inclusive LCA model and our final multivariable model. Our data were self-reported, which is subject to social desirability bias. Our use of computer-assisted self-interviews may have minimized this bias. Notably, our questionnaire asked participants to report their lifetime prevalence of chemsex engagement; it should not be interpreted as recent chemsex engagement. Nonetheless, participants who reported general use instead of chemsex-related use may still be subject to misclassification bias. Future research should utilize a more nuanced measure, such as the duration of regular substance use and chemsex-related substance use, for measure validation and to provide further insights into their overlap to evaluate the impact of chemsex on chronic substance use. Lastly, the cross-sectional nature of our study does not address potential changes in substance-using patterns, sexual behaviors, and HIV/STI statuses over time, nor does it establish causality between these variables and latent class membership.

### Conclusions

Our findings suggest that different strategies should be employed to target specific subgroups of MSM who use various substances. For MSM who primarily engage in the inhalation and injection of crystal meth before or during sex, interventions should focus on increasing their PrEP coverage to lower their HIV transmission risks. For MSM who engage in alcohol consumption and a range of various substances before or during sex, interventions should focus on increasing their HIV risk perceptions and PrEP awareness while scaling up their HIV testing uptake by engaging them via HIV self-testing and online platforms (Samoh et al., 2021). MSM in Thailand are experiencing a high burden of multiple health disparities related to HIV risk and substance use. Tailored interventions are urgently needed to reverse health trajectories.

## Supplementary Information

Below is the link to the electronic supplementary material.

Supplementary file1 (DOCX 94 kb)

## Data Availability

Data are available upon request to the corresponding author at thomas.gua@mahidol.edu (Thomas E. Guadamuz).
