# Supplementary material for: Patterns of Chemsex Substance Use and Its Association with HIV Transmission Risk Among Men Who Have Sex with Men in Thailand: A Latent Class Analysis
Source: Arch Sex Behav. 2024 Aug 16;53(9):3527–36. doi: 10.1007/s10508-024-02868-8 (PMC11390813; doi:10.1007/s10508-024-02868-8)
Supplement: Supplementary file 1 — Supplementary file1 (DOCX 94 kb) [file 10508_2024_2868_MOESM1_ESM.docx]

| Supplementary Table 1. Goodness-of-fit indices comparing class membership models of substance use before or during sex patterns | | | | | | | | | | | |  |  |  |
| --- | --- | --- | --- | --- | --- | --- | --- | --- | --- | --- | --- | --- | --- | --- |
| Number of Classes | Npar | LL | BIC | | aBIC | | CAIC | AWE | | | RE | SC% |  |  |
| Class-2 | 16 | -2487.07 | 5074.57 | | 5023.78 | | 5090.57 | 5222.99 | | | NA | 1.00 |  |  |
| Class-3 | 33 | -2344.70 | 4896.52 | | 4791.77 | | 4929.52 | 5202.65 | | | 0.73 | 0.43 |  |  |
| Class-4 | 50 | -2256.89 | 4827.61 | | 4668.90 | | 4877.61 | 5291.45 | | | 0.79 | 0.08 |  |  |
| Class-5 | 67 | -2195.30 | 4811.13 | | 4598.45 | | 4878.13 | 5432.67 | | | 0.82 | 0.09 |  |  |
| Class-6 | 84 | -2157.62 | 4842.47 | | 4575.83 | | 4926.47 | 5621.71 | | | 0.86 | 0.04 |  |  |
| Class-3 (inclusive) | **74** | **-2110.84** | **4686.15** | | **4451.25** | | **4760.15** | **5372.62** | | | **0.82** | **0.14** |  |  |
| Npar: number of parameters; LL: log likelihood ratio; BIC: Bayesian Information Criteria; aBIC: sample-size adjusted BIC; CAIC: Consistent Akaike Information Criteria; AWE: Approximate Weight of Evidence Criteria; RE: Relative Entropy; SC%: smallest class percentage | | | | | | | | | | | |  |  |  |
| Supplementary Table 2. Univariate multinomial logistic regression examining participants’ socio-demographics and behavioral characteristics in relation to substance use before or during sex class membership (reference= negligible sexualized substance users) | | | | | | | | | | | | | | |
|  | | | | Sexualized substance users | | | | | Exclusive chemsex users | | | | | |
|  | | | | OR | | 95% CI | | | OR | 95% CI | | | | |
| Age | | | |  | |  | | |  |  | | | | |
| 18-29 | | | | (Ref) | |  | | | (Ref) |  | | | | |
| 30-39 | | | | 0.25 | | 0.16 – 0.39 | | | 0.20 | 0.11 – 0.37 | | | | |
| 40+ | | | | 0.66 | | 0.41 – 1.09 | | | 0.16 | 0.06 – 0.40 | | | | |
|  | | | |  | |  | | |  |  | | | | |
| Employment | | | |  | |  | | |  |  | | | | |
| Full-time | | | | (Ref) | |  | | | (Ref) |  | | | | |
| Part-time | | | | 0.90 | | 0.51 – 1.58 | | | 1.23 | 0.56 – 2.7 | | | | |
| Student/Unemployed/Others | | | | 1.38 | | 0.90 – 2.12 | | | 2.27 | 1.28 – 4.01 | | | | |
|  | | | |  | |  | | |  |  | | | | |
| Education | | | |  | |  | | |  |  | | | | |
| None - Secondary | | | | (Ref) | |  | | | (Ref) |  | | | | |
| Tertiary or above | | | | 0.69 | | 0.42 – 1.12 | | | 0.06 | 0.03 – 0.12 | | | | |
|  | | | |  | |  | | |  |  | | | | |
| Monthly Income | | | |  | |  | | |  |  | | | | |
| Less than 100USD | | | | 1.87 | | 0.99 – 3.54 | | | 16.99 | 4.79 – 60.29 | | | | |
| 101-450USD | | | | 23.59 | | 8.31 – 67.02 | | | 1.03 | 0.69 – 1.54 | | | | |
| More than 450USD | | | | (Ref) | |  | | | (Ref) |  | | | | |
|  | | | |  | |  | | |  |  | | | | |
| Sexual orientation | | | |  | |  | | |  |  | | | | |
| Gay | | | | 0.42 | | 0.23 – 0.79 | | | 5.20 | 0.68 – 39.65 | | | | |
| Bisexual | | | | (Ref) | |  | | | (Ref) |  | | | | |
|  | | | |  | |  | | |  |  | | | | |
| Attended a private sex party or circuit party in past 3 years | | | |  | |  | | |  |  | | | | |
| Private sex party | | | | (Ref) | |  | | | (Ref) |  | | | | |
| Circuit party | | | | 0.26 | | 0.13 – 0.54 | | | 0.14 | 0.03 – 0.57 | | | | |
|  | | | |  | |  | | |  |  | | | | |
| Ever tested for HIV | | | |  | |  | | |  |  | | | | |
| Ever | | | | (Ref) | |  | | | (Ref) |  | | | | |
| Never | | | | 1.75 | | 0.97 – 3.16 | | | 0.35 | 0.20 – 0.59 | | | | |
|  | | | |  | |  | | |  |  | | | | |
| HIV status | | | |  | |  | | |  |  | | | | |
| Negative | | | | (Ref) | |  | | | (Ref) |  | | | | |
| Positive | | | | 0.20 | | 0.05 – 0.89 | | | 2.31 | 1.39 – 3.83 | | | | |
| Don’t know | | | | 1.39 | | 0.79 – 2.47 | | | 0.53 | 0.33 – 0.86 | | | | |
|  | | | |  | |  | | |  |  | | | | |
| Any STI in the past 12 months | | | |  | |  | | |  |  | | | | |
| Yes | | | | 1.73 | | 1.00 – 2.99 | | | 2.74 | 1.86 – 4.03 | | | | |
| No | | | | (Ref) | |  | | | (Ref) |  | | | | |
|  | | | |  | |  | | |  |  | | | | |
| Number of sexual partners in the past 6 months | | | |  | |  | | |  |  | | | | |
| 0-1 | | | | (Ref) | |  | | | (Ref) |  | | | | |
| 2-5 | | | | 8.36 | | 2.50 – 27.91 | | | 1.39 | 0.87 – 2.23 | | | | |
| 6+ | | | | 22.96 | | 6.03 – 87.46 | | | 8.60 | 4.45 – 16.61 | | | | |
|  | | | |  | |  | | |  |  | | | | |
| Condom use in last anal intercourse | | | |  | |  | | |  |  | | | | |
| Yes | | | | (Ref) | |  | | | (Ref) |  | | | | |
| No | | | | 16.32 | | 6.75 – 39.48 | | | 4.54 | 3.04 – 6.78 | | | | |
|  | | | |  | |  | | |  |  | | | | |
| Ever had group sex | | | |  | |  | | |  |  | | | | |
| Never - Sometimes | | | | (Ref) | |  | | | (Ref) |  | | | | |
| Often | | | | 1.61 | | 0.79-3.27 | | | 0.81 | 0.53-1.23 | | | | |
|  | | | |  | |  | | |  |  | | | | |
| Ever received things or opportunities in exchange for sex | | | |  | |  | | |  |  | | | | |
| Yes | | | | 35.83 | | 15.26 – 84.11 | | | 3.72 | 2.43 – 5.67 | | | | |
| No | | | | (Ref) | |  | | | (Ref) |  | | | | |
|  | | | |  | |  | | |  |  | | | | |
| Ever provided things or opportunities in exchange for sex | | | |  | |  | | |  |  | | | | |
| Yes | | | | 54.75 | | 18.95 – 158.20 | | | 6.14 | 4.05 – 9.31 | | | | |
| No | | | | (Ref) | |  | | | (Ref) |  | | | | |
|  | | | |  | |  | | |  |  | | | | |
| Ever meet sexual partners online | | | |  | |  | | |  |  | | | | |
| Never-occasionally | | | | (Ref) | |  | | | (Ref) |  | | | | |
| Often | | | | 37.95 | | 13.24 – 108.79 | | | 2.65 | 1.80 – 3.92 | | | | |
|  | | | |  | |  | | |  |  | | | | |
| Heard of PrEP (n=424) | | | |  | |  | | |  |  | | | | |
| Yes | | | | 0.65 | | 0.41 – 0.85 | | | 3.14 | 1.46 – 6.75 | | | | |
| No | | | | (Ref) | |  | | | (Ref) |  | | | | |
|  | | | |  | |  | | |  |  | | | | |
| Taken PrEP (n=424) | | | |  | |  | | |  |  | | | | |
| Yes | | | | 0.14 | | 0.03 – 0.62 | | | 3.68 | 2.24 – 6.07 | | | | |
| No | | | | (Ref) | |  | | | (Ref) |  | | | | |
|  | | | |  | |  | | |  |  | | | | |
| Want to take PrEP (n=314) | | | |  | |  | | |  |  | | | | |
| Yes | | | | 5.85 | | 1.72 – 19.97 | | | 1.09 | 0.58 – 2.04 | | | | |
| No | | | | (Ref) | |  | | | (Ref) |  | | | | |
| AOR=adjusted odds ratio, CI=confidence interval | | | | | | | | | | | | | | |
| Supplementary Figure 1. Elbow plot for LCA model class renumeration | | | | | | | | | | | | | |  |
| 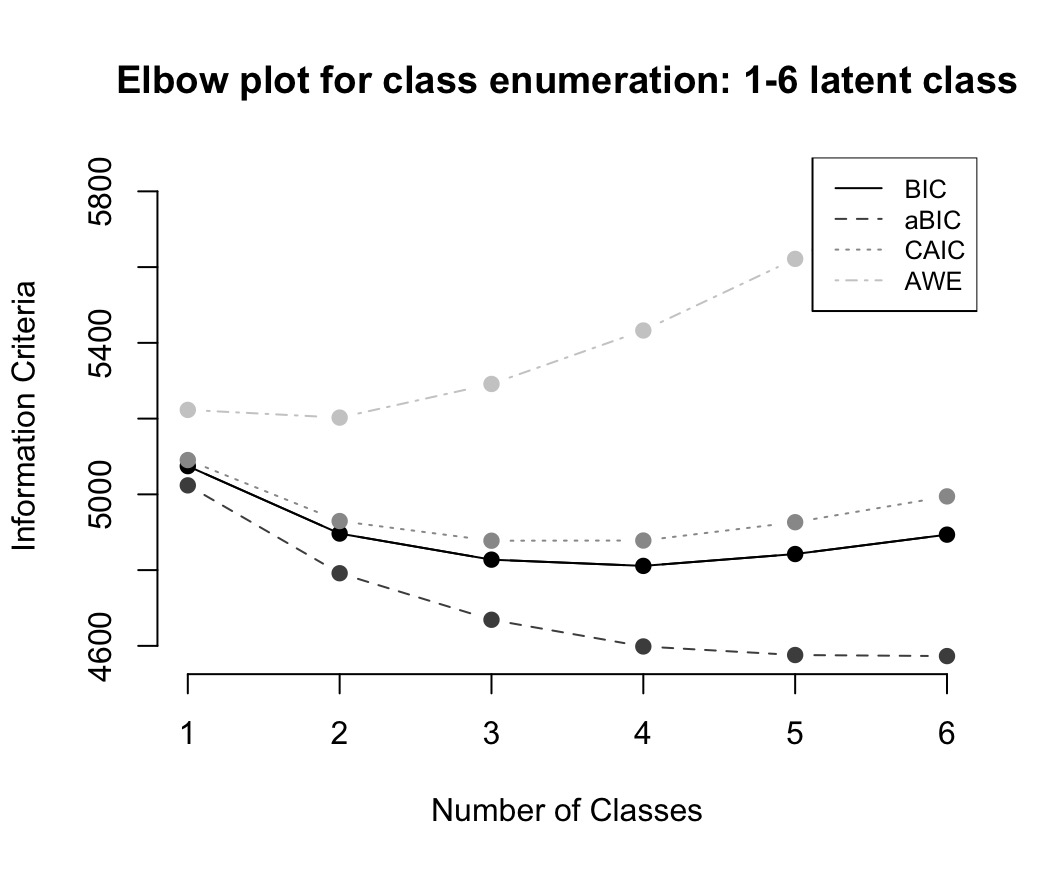 | | | | | | | | | | | | | |  |
